# Effects of Graphite Oxide Nanoparticle Size on the Functional Properties of Layer-by-Layer Coated Flexible Foams

**DOI:** 10.3390/nano11020266

**Published:** 2021-01-20

**Authors:** Lorenza Maddalena, Julio Gomez, Alberto Fina, Federico Carosio

**Affiliations:** 1 Dipartimento di Scienza Applicata e Tecnologia, Politecnico di Torino, Alessandria Campus, Viale Teresa Michel 5, 15121 Alessandria, Italy; lorenza.maddalena@polito.it (L.M.); alberto.fina@polito.it (A.F.); 2 Avanzare Innovacion Tecnologica S.L., Avda. Lentiscares 4-6, Poligono Industrial Lentiscares, 26370 Navarrete, Spain; julio@avanzare.es

**Keywords:** chitosan, graphite oxide, layer by layer, flame retardancy

## Abstract

The exploitation of self-assembled coatings comprising graphite oxide (GO) nanoplates has been recently demonstrated as a promising route to improve the fire safety of flexible polyurethane (PU) foams. However, limited knowledge has been gathered on the correlations between the physical and chemical properties of different GO grades and the performance obtained in this application. This work addresses the effects of the nanoparticle dimensions on the layer-by-layer (LbL) assembly and flame-retardant properties of GO-based coatings deposited on PU foams. To this aim, three GO bearing different lateral sizes and thicknesses were selected and LbL-assembled with chitosan (CHIT). Coating growth and morphology were evaluated by FTIR and FESEM, respectively. The resulting CHIT/GO assemblies were demonstrated to be capable of slowing down the combustion of the PU both in flammability and forced combustion tests. In addition, compressive stress/strain tests pointed out that the LbL-coated foams (22–24 kg/m^3^) could easily replace denser commercial PU foam (40–50 kg/m^3^) with weight reduction potentials in the transport field. These results are correlated with the properties of the employed GO. The production of assemblies characterized by a high density of CHIT/GO interfaces is identified as the main parameter controlling the FR efficiency and the mechanical properties of the coatings.

## 1. Introduction

**Figure 1:**
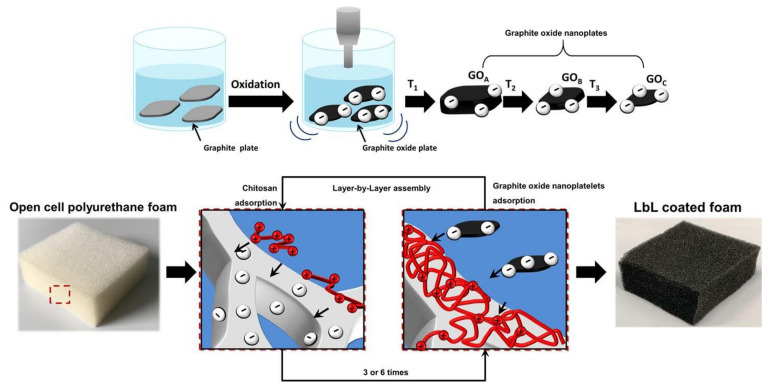
Schematic representations of the preparation process of graphite oxide nanoplates with different sizes and the subsequent layer-by-layer (LbL) assembly on polyurethane (PU) foams.

Open cell foamed polyurethane (PU) is one of the most employed solutions for the production of goods ranging from sound proofing panels to thermal insulation [1]. In particular, PU foams are the main component of upholstered furniture such as matrasses and automotive seats [2]. However, PU foams are highly flammable and when subjected to a small flame or a heat flux they can ignite and self-sustain the flame while releasing potentially toxic gasses [3]. Moreover, during the combustion of PU foam items, the release of flaming droplets may easily ignite other flammable materials in proximity, quickly spreading the fire in the room. Thus, the flammability of PU foams strongly limits their application in many fields, such as transport and construction where fire safety must be guaranteed. To address this problem, flame-retardant PU foams are commonly prepared by the use of flame-retardant additives in the polymer formulation. Unfortunately, this might alter the foam production process, leading to reductions in the final open cell structures and the need for different foaming conditions [4,5]. As an alternative to FR inclusion in the PU formulation, a post treatment of the foam by means of surface coating has recently been proved to confer the required flame-retardant properties to the foam, without altering the foam production processing [6,7,8]. Indeed, the surface of a polymer may play a key role during its combustion as it is through the surface that heat is transferred from the flame to the bulk and the combustible volatiles are released to feed the combustion processes [9]. For example, thin-nanostructured coatings deposited by the layer-by-layer assembly technique (LbL) can be exploited to control the abovementioned heat and mass exchanges [10,11,12]. The LbL is a well-known surface deposition technique based on the multistep adsorption of oppositely charged polyelectrolytes or nanoparticles from aqueous media. The assembly is generally driven by electrostatic attraction but other interactions, such as hydrogen bonding, Van der Waals and hydrophobic interactions can be exploited [13,14]. Moreover, the thickness of the coating can be tuned by controlling the deposition parameters such as the pH [15], the nature of the polyelectrolytes and their concentration [16,17], the temperature [18] and the ionic strength [19,20], making this technique very versatile. By combining polyelectrolytes and nanoplates, such as clay- or graphene-related materials, it is possible to deposit nanostructured coatings that exhibit a “brick and mortar” fashion where nanoparticles are embedded in a polymer matrix [21,22,23,24,25]. This particular structure has been proved to significantly increase the flame retardancy of PU foams due to a combined effect of heat shielding/reradiating and delayed release of combustible volatiles [25,26]. Although most of the developed coatings have been focused on the exploitation of mineral nanoplates such as montmorillonite [25], vermiculite [27] and kaolinite [28], recent reports showed that oxidized graphitic materials can endow superior flame-retardant properties [21]. Indeed, it has been demonstrated that an LbL coating comprising of six bi-layers (BLs) alternating chitosan (CHIT) and graphite oxide (GO) nanoplates can improve the flame retardancy of PU foams by limiting the flaming ignition in forced combustion tests and suppressing the melt-dripping phenomenon during flammability tests [22]. These results were explained by pointing out how the char-forming abilities of CHIT, coupled with GO, resulted in the build-up of a thermally stable barrier. In addition, the high aspect ratio of GO allowed for an efficient overlap of nanoplates in a functional brick and mortar structure capable of slowing down the release of volatiles towards the flame. However, despite the previous work demonstrating the FR effectiveness of GO-based LbL coatings on PU foam, there is still limited knowledge on how graphene related materials properties such as thickness, lateral size, O/C ratio, etc., which are affected by the preparation routes [29,30,31,32], can influence the FR performances. Thus, in the present paper, we aim at further investigating the potentialities of GO-based FR coatings by evaluating the effects of different GO dimensions. To this aim, three GO suspensions having different lateral size and thicknesses were prepared by modified Hummers’ method and then LbL assembled with CHIT (Figure 1) on PU foams and their flame-retardant properties were evaluated to establish a correlation between GO size and FR performance.

## 2. Materials and Methods

Polyurethane foams (density 20 kg/m^3^ and thickness of 18 mm) were purchased from the local warehouse. Prior to the LbL deposition the foams were cut and washed with deionized water in order to remove dust and completely open the porous structure. After the washing step, the foam was dried to constant weight in a ventilated oven (80 °C). Chitosan (CHIT, 75–85% deacetylated), acetic acid, poly(acrylic acid) (PAA, solution average Mw ~100,000 g/mol, 35 wt% in H_2_O) and branched poly(ethylene imine) (BPEI, Mw ~25,000 g/mol by Laser Scattering, Mn ~10,000 g/mol by Gel Permeation Chromatography, as reported in the material datasheet) were purchased from Sigma-Aldrich (Milano, Italy). Then, 1.0 wt% water suspension of GO with different average lateral sizes was prepared using a modified Hummers’ method in H_2_SO_4_ as described in Supplementary Material. The nanoplates, obtained after different sonication times (30, 60, 120 min), were labelled as GO_A_, GO_B_ and GO_C_, respectively. A Q20 Millipore system (Milano, Italy) was used to supply ultrapure (18.2 MΩ and pH 5.50 ± 0.01) employed for the preparation of solutions adopted in this work. A single side-polished (100) Si wafer was used as model substrate for monitoring the LbL growth. Viscosity of GO suspensions has been determined using a rotational viscometer Brookfield EVO Expert R, employing the low-viscosity-adapter tool, in a thermostatic bath to ensure constant temperature during the measurement (25.0 °C ± 0.1). Measurements were carried out in triplicate, and the average results are reported with their experimental deviation.

### 2.1. Layer-by-Layer Deposition

The surface of model substrate Si wafers was primed by 0.1 wt% BPEI (10 min dipping) and 1 wt% PAA solution (10 min dipping), aiming at replicating the same PAA activating procedure employed on PU foam. After these two steps, the LbL assembly was performed by alternately dipping the Si wafer into the 0.25 wt% CHIT and 0.5 wt% GO baths. The first BL (i.e., one CHIT/GO pair) was deposited using 10 min as deposition time, while for subsequent BL, the time was reduced to 1 min. A washing step is performed after each deposition by dipping the substrate in ultrapure water (1 min). The Si wafer is then dried using compressed air (oil free). IR spectra were collected after each drying step, the growth was evaluated up to 10 BL.

PU foams were activated by 1 wt% PAA solution (10 min). After the activation step, PU foams were alternatively immersed into the positively (CHIT) and negatively (GO) charged baths. A rinsing step is performed after each deposition by ultrapure water. No drying step is performed between each deposition/washing step. In order to let the solution/suspension or washing water penetrate inside the open structure of the foams, the foams were squeezed several times while submerged into each bath. The dipping times were the same employed for the model Si wafer. The process was employed to deposit 3 and 6BL for each system. The foams are dried at the end of the deposition by placing them in a ventilated oven (80 °C). The coating weight gain % was evaluated by weighting the dry foams before and after the deposition.

### 2.2. Characterization

The LbL growth of the assembly was monitored by FTIR spectroscopy (Perkin-Elmer Frontier, 32 scansions, 4 cm^−1^ resolution, Waltham, MA, USA). The coating cross-section on Si wafer was imaged by a high-resolution Field Emission Scanning Electron Microscopy (FESEM, Zeiss Merlin 4248, beam voltage: 5 kV, Oberkochen, Germany). Samples were chromium-coated prior to FESEM observations.

The surface morphology of untreated and LbL-treated PU foams was investigated using a LEO-1450VP Scanning Electron Microscope (SEM, Zeiss Merlin, beam voltage: 5 kV, Jena, Germany). Foams were sampled by cutting small pieces (1 cm^3^) from the transversal section of the foams. The produced specimens were posited on conductive adhesive tapes and gold-metallized prior to SEM imaging.

Flammability tests were performed in horizontal configuration by applying a 20 mm pre-mixed blue methane flame on the short side of the foam specimen (50 × 150 × 15 mm) positioned on a metallic grid, following the position setup described by ASTM D 4986 standard. The flame application time was set to 3 s. Three specimens were tested for each formulation. The flame spread rate, the occurrence of the melt dripping phenomenon and the final residue were evaluated. Forced combustion behavior was evaluated by an oxygen consumption cone calorimeter (Fire Testing Technology, FTT, East Grinstead, England) under 35 kW/m^2^ radiative heat flux, following the ISO 5660 standard. The specimen size was 50 × 50 × 18 mm. Samples were wrapped in double-folded aluminum foil leaving only the surface directly exposed to the heat flux uncovered. The wrapped sample is positioned on the sample holder with its rear face supported by two ceramic backing pads having the same dimensions (i.e., 50 × 50 mm). A spark igniter is used to ignite the combustible volatile gasses released by the sample upon exposure to the cone heat flux. Four specimens were tested for each formulation. The parameters evaluated were: time to ignition (TTI), peak of heat release rate (pkHRR), total heat release (THR), total smoke release (TSR) and final residue. Prior to flammability and cone calorimetry tests, all specimens were conditioned in a climatic chamber (48 h at 23.0 ± 0.1 °C and 50.0 ± 0.1% R.H.). The flame-retardant efficiency of the coatings at different BL numbers, with respect to both heat and smoke parameters, was calculated as a dimensionless number by evaluating the ratios pkHRRreduction %/weight gain % and TSRreduction %/weight gain %, respectively. Mechanical properties were evaluated by compression test conducted on a dynamometer (Instron 5966, 2 kN cell, Canton, MA, USA) stacking 2 samples of 30 × 30 × 18 mm between two horizontal plates and following the EN ISO 2439 standard (60% compression, deformation speed 100 mm/min). The firmness was calculated as the stress at 40% deformation, according to ISO 3386 standard. Prior to the tests, samples were conditioned 23.0 ± 0.1 °C for 48 h at 50.0 ± 0.1% R.H. in a climatic chamber.

## 3. Results

### 3.1. Nanoplate Dimensions Trend, Layer-by-Layer Growth and Characterization

The dimensions for nanoplates GO_A_, GO_B_ and GO_C_ were characterized combining viscosity and laser diffraction measurements, as summarized in Table 1.

The laser diffraction (LD) technique has been extendedly used for the characterization of GO and other graphene material dispersions. In comparison, the results of lateral size determination by using other characterization techniques such as SEM, AFM, TEM or optical microscopy show good correlation [33,34]. LD measurements (Table 1) suggest a decreasing lateral size with the increase in sonication time, based on the D50 values equal to 61 ± 2, 39 ± 2 and 34 ± 3 µm for GO_A_, GO_B_ and GO_C_, respectively, attributed to the fragmentation during ultrasonication [35]. The fragmentation effect promoted by ultrasonication is also evidenced by pH measurements, which underline how smaller nanoplates can expose a higher concentration of acidic functionalization for the same GO concentration, resulting in a trend of acidity GO_C_ > GO_B_ > GO_A_. As far as the aspect ratio is concerned, it has been demonstrated that the viscosity of diluted graphene oxide suspension is dependent on the nanoplates dimensions, for a given volume concentration of particles [36]. By comparing suspensions with the same wt% concentration it is possible to observe a trend where viscosity increases (Table 1) by reducing the lateral size of the suspended GO. Thus, combining the parameters reported in Table 1 with previously reported literature on GO suspensions [34,37], it is possible to devise a trend where the aspect ratio increases by increasing the treatment time (GO_C_ > GO_B_ > GO_A_).

**Figure 2:**
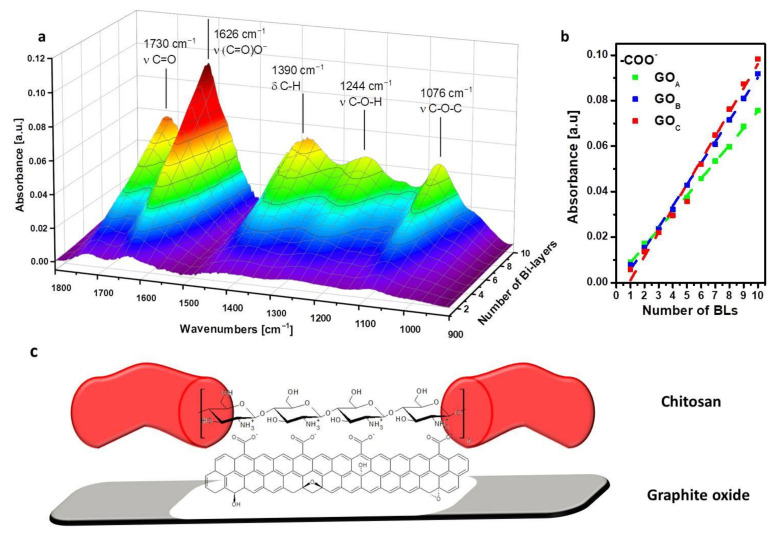
FTIR LbL growth of CHIT/GO_A_ assembly conducted on a silicon wafer as substrate (**a**), 1626 cm^−1^ plot as function of deposited bi-layer (BL) for all the under-study assemblies (**b**), CHIT/GOx (**c**) assemblies.

The different GO grades were coupled with CHIT and assembled on a silicon wafer as model substrate monitoring the growth of the coatings by FTIR spectroscopy. Figure 2 reports the 3D plot of the spectra acquired at each deposition step for the GO_A_/CHIT system, the intensity of the peak ascribed to the carboxylate groups (at 1626 cm^−1^) as a function of BL number for each of the coatings obtained with the different GO grades, as well as a schematic proposed interaction between CHIT and GO. The spectra of neat CHIT and GO with the signal attributions, the 10BL GO_B_/CHIT and 10BL GO_C_/CHIT are reported in Figures S1 and S2.

The neat GO shows characteristic signals at 1725, 1621 and 1054 cm^−1^, assigned to the stretching modes for C=O in carboxylic acid, COO^−^, and C-O, respectively [38]. Hydroxyl groups are also visible in the broad range between 3800 and 3000 cm^−1,^ owing to H-bonding. As far as CHIT is concerned, C-H stretching vibrations are visible at 2900 and 2880 cm^−1^ for asymmetric and symmetric stretching of CH_2_, respectively [39]. The most intense band of CHIT is located at 1080 cm^−1^ and is related to the stretching vibrations of the C-O-C group in the glycosidic linkage [40]. When CHIT is coupled with the different GO under study, the resulting LbL assembly follows a step-by-step increase in the characteristic signals of both components as a function of the deposited BL number (Figure 2a, Figure S2). The assembly is driven by the electrostatic interactions taking place between the GO -COO^−^ and CH -NH_3_^+^ functional groups, as also supported by previous studies [41].

**Figure 3:**
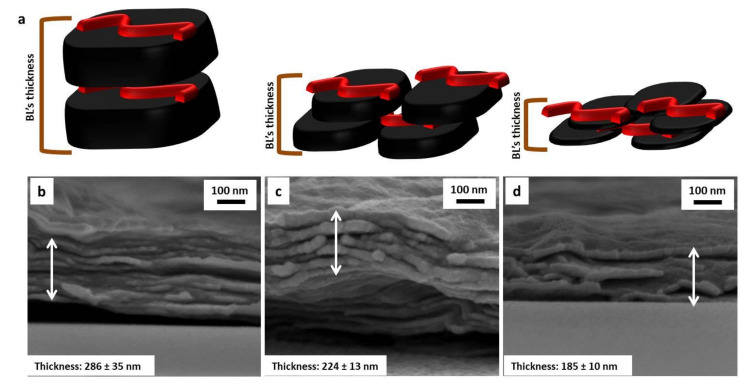
Schematic representation of 1BL CHIT/GO_A_ (left), 1BL CHIT/GO_B_ (center) and 1BL CHIT/GO_C_ (right) thickness (**a**). FESEM micrograph of 10BL CHIT/GO_A_ (**b**), 10BL CHIT/GO_B_ (**c**), and 10BL CHIT/GO_C_ (**d**) coatings on Si wafer cross-section.

By plotting the signal at 1626 cm^−1^, ascribed to the –COO^−^ stretching vibration mode of GO vs. BL number (Figure 2b), a linear regime growth is evidenced for each system. This finding is in accordance with previously reported LbL coatings encompassing CHIT and GO [22]. In addition, while at low BL number, there are almost no differences between the systems; after five deposited BL, the intensity growth of the –COO^−^ signal appears to follow the trend GO_A_ < GO_B_ < GO_C_. This suggests an effect of GO size on the coating thicknesses and/or on a higher carboxylate group concentration within the coating. To verify the coating thickness, the cross-sections of 10BL coatings have been imaged by FE-SEM (Figure 3). 

The deposited assemblies consist of a dense, continuous and layered structure where the GO nanoplates are embedded within the CHIT matrix. As observable in the collected micrographs, the thicknesses of 10BL coatings were measured in the range of 286 ± 35 nm, 224 ± 13 nm and 185 ± 10 nm for CHIT/GO_A_, CHIT/GO_B_ and CHIT/GO_C_ assemblies, respectively. Therefore, differences observed in FTIR signals appear to be related to the concentration of the functional groups on GO rather than the coating thickness. Indeed, as suggested by pH values for the GO suspensions, the different sonication times promoted the exposition of a higher number of acidic functions (Table 1). Thus, the thinner flakes of GO_C_ may promote the interaction of a higher number of –COO^−^ with –NH_3_^+^ groups of CHIT with respect to the larger and thicker GO_A_. The GO_B_ results in an intermediate situation. This produces the observed trend when plotting the intensity of COO^−^ signals vs. BL number as reported in Figure 2b pointing out that, with respect to CHIT/GO_A_, the CHIT/GO_C_ achieves a greater number of interactions (i.e., chitosan/GO interfaces) per assembly thickness.

### 3.2. Morphology on PU Foams

**Figure 4:**
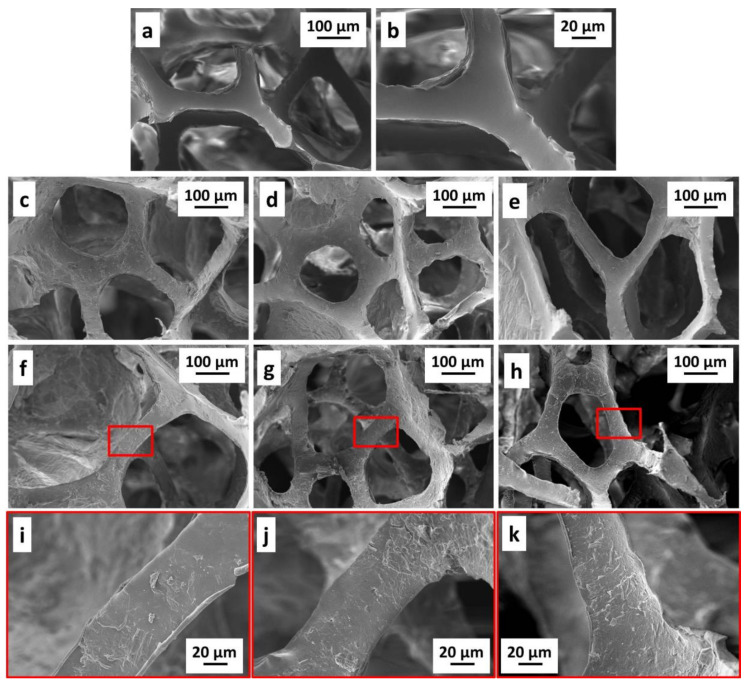
SEM micrograph of: (**a**) and (**b**) neat PU foam; 3BL (**c**) GO_A_, (**d**) GO_B_, (**e**) GO_C_; 6BL (**f**) GO_A_, (**g**) GO_B_, (**h**) GO_C_; high magnification micrograph of 6BL (**i**) GO_A_, (**j**) GO_B_, (**k**) GO_C_.

Assemblies exploiting the different GO grades were deposited on PU foams and the changes in surface morphology evaluated by SEM. The collected micrographs of untreated and LbL-treated foams are reported in Figure 4.

The neat PU foam is characterized by an open cell structure with continuous and smooth surfaces with no significant irregularities evidenced by SEM micrographs (Figure 4a,b).

The LbL deposition of 3BL modifies the cell wall morphology of the PU foams while retaining its original open cell structure (Figure 4c–e). By increasing the number of deposited layers, the coatings became more wrinkled and irregular, owing to the increased number of GO nanoplates embedded in the assembly (see Figure 4f–k). The weight gain associated to the LbL deposition (Table 2) follows the same trend related to the GO dimensions (i.e., GO_A_ > GO_B_ > GO_C_) and it is in accordance with the morphological evaluation performed on the coating cross-sections (Figure 3).

### 3.3. Flame-Retardant Properties

**Figure 5:**
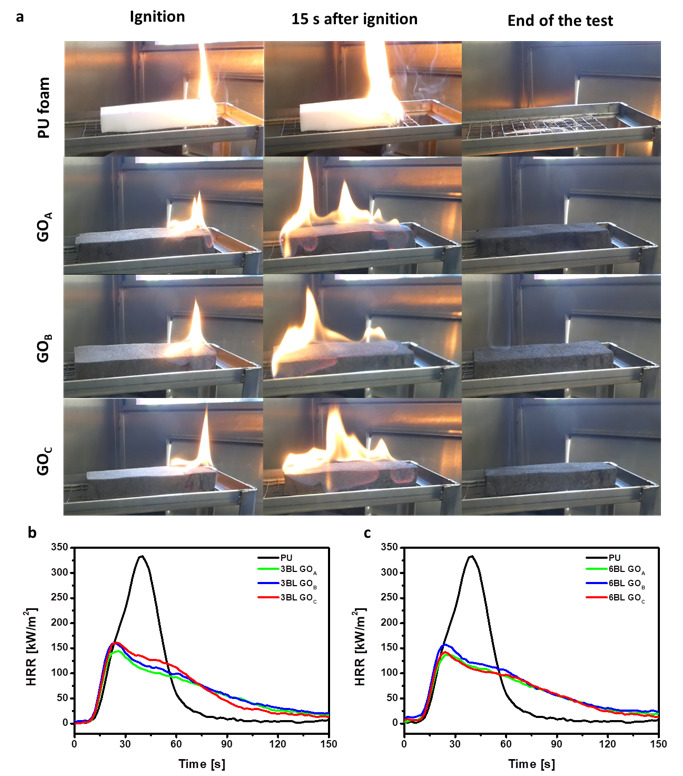
Flame retardant characterization: (**a**) Pictures of flammability test in horizontal configuration of untreated PU foam, 3BL CHIT/GO_A_ treated PU foam, 3BL CHIT/GO_B_ PU foam and 3BL CHIT/GO_C_ PU foam. First column: right after ignition, second column: 15 s after ignition and third column: end of the test. (**b**) HRR vs time plots of 3BL samples. (**c**) HRR vs time plots of 6BL samples.

The flame-retardant properties of the treated foams were evaluated by flammability and cone calorimetry tests. These tests provide complementary information related to the foam behavior after the exposure to a small flame or a radiative heat flux. The aim is to establish whether the GO dimensions can affect the FR performance of the coatings. Figure 5a reports snapshots from flammability tests on pristine and coated foams, whereas Figure 5b,c show heat release rate vs. time plots from cone calorimetry. Table 2 summarizes the calculated parameters from each test.

When exposed to a small flame, the neat PU foam ignites immediately and is quickly consumed by the flames. Moreover, during combustion, drops of molten polymer fall down and ignite the cotton below the sample. This phenomenon is called melt dripping and represents an additional threat of PU foams as the flaming droplets can easily spread the fire to other materials.

LbL-treated foams display a different behavior: after ignition, the flame spreads along the edges and the upper surface of the foams, travelling the entire length of the sample and then extinguishes, leaving a coherent residue that maintains the original shape of the foam. The melt-dripping phenomenon is completely suppressed, thus reducing flame propagation risks to other items in a real fire scenario. By comparison with the unmodified foam, 3BL samples show an increased flame spread rate (Table 2), while the same parameter is reduced when the BL number is increased to 6. By comparing the final residues collected at the end of the test with the coating weight gain (Table 2) it is possible to highlight that although the flame spreads to the entire surface of the sample, the PU foam is not completely decomposed and volatilized and the flame extinguishes before being able to completely consume the whole foam. Indeed, post combustion residue investigations demonstrated that only the first few mm of the surface exposed to the flame were actually decomposed while the remaining portion of foam was left undamaged. This is ascribed to the presence of the coating that limits the release of volatiles, while preventing the collapse of the foam and protecting the underlying material. To further investigate the combustion process, forced combustion tests were conducted, in a condition representative of an early stage developing fire. Figure 5b,c report HRR curves vs. time plots of untreated and LbL-coated foams, while cone calorimetry parameters are collected in Table 2. After ignition, the pristine PU quickly collapses, producing a pool of low viscosity liquid that vigorously burns reaching a maximum heat release rate (pkHRR) in the range of 300 kW/m^2^. When 3BL is deposited, the burning behavior of the foam changes: the structural collapse is prevented and the peak of heat release is considerably decreased by approximately 50% for 3BL CHIT/GO_A_, 3BL GO_B_- and 3BL CHIT/GO_C_ (Table 2). The 6BL samples showed only slight improvements in performances, compared to 3BL, evidencing that thicker deposited layers do not provide better performance in terms of pkHRR reduction. As far as the total smoke release is concerned, the presence of both 3BL and 6BL coatings dramatically decrease TSR values, as reported in Table 2. The CHIT/GO_A_ assembly exhibits the best performances in terms of TSR reduction producing a 69% and 77% for 3BL and 6BL, respectively (Table 2). The other two assemblies still produce significant reductions in the 56–67% range. The collected HRR and smoke data suggest a similar FR behavior for the three CHIT/GO systems under study. However, it is worth highlighting that these results are achieved with rather different coating weight gains, indicating a different coating efficiency. 

**Figure 6:**
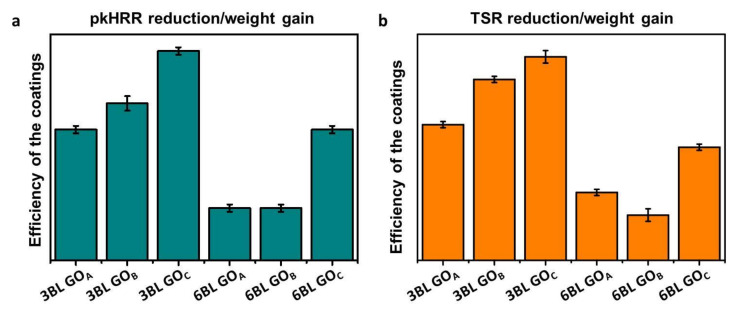
Coating flame-retardant efficiency as function of pkHRR reduction/weight gain (**a**) and TSR reduction/weight gain (**b**) for 3BL- and 6BL-treated foams.

To take into account the coating thickness, an efficiency parameter was calculated by normalizing the observed reductions in pkHRR and TSR by the coating weight gain ascribed to each CHIT/GO system. The resulting values are reported in Figure 6.

Interestingly, within each coated foam at a given BL number, the higher efficiency is constantly obtained by GOc. This behavior can be explained by considering that, due to the reduced thickness and lowest lateral size, the GO_C_ can produce an LbL assembly where the through-thickness density of interfaces and nanoparticle overlapping are maximized. This results in a more efficient brick and mortar structure capable of better control of the mass and heat exchange between the flame and the PU. These observations clearly point out the greater importance of achieving an optimal nanostructuration over the total coating weight gain.

### 3.4. Mechanical Behavior of LbL-Treated Foams

**Figure 7:**
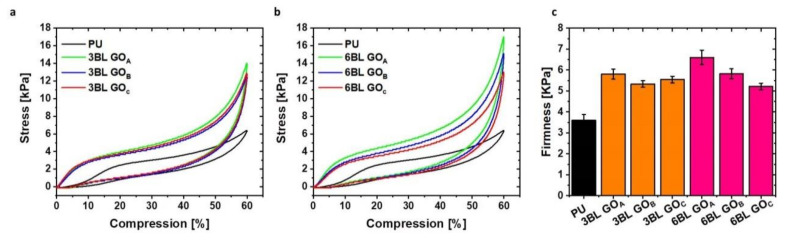
Mechanical compression test performed on 3BL (**a**) and 6BL (**b**) treated PU foams. Firmness comparison of different treated samples (**c**).

The mechanical properties of the neat and LbL-treated foams were also assessed by compression test following the EN ISO 2439 standard. Figure 7 reports the fourth cycle stress/compression curves for neat, 3BL- and 6BL-treated foams along with a graphical representation of the calculated firmness.

From an overall point of view, an untreated open cell PU foam shows a three-phase compressive/stress curve [2]. In the first phase, under 10% of compression, the foam acts as a linear elastic material with the walls of the PU foam proving simple resistance to loads. In the second phase, between 15 and 40% deformation, the walls of the cellular structure suffer progressive buckling, thus becoming softer and eventually reach the last phase where the buckling is complete, and the stress increases steeply. The third phase of the stress–strain curve is normally referred as the densification stage because the entrained gas in the foam is mostly expelled by the compression.

While a similar behavior is observed for all the LbL-treated foams, the stress values are significantly higher than those measured for the unmodified foam. This can be ascribed to the presence of the coating that, thanks to the strong ionic interactions occurring between CHIT and GO, acts as a rigid exoskeleton providing higher stiffness to the foam. Notwithstanding this, the treated foams still maintain the load/unload hysteresis curve typical of flexible PU and thus the ability of recovering the initial shape after being cyclically deformed, which is crucial for the typical application of flexible PU foams. The observed behavior can be related to a strong coating adhesion and homogeneity through the entire thickness of the foam. This is also corroborated by previous findings on nanocellulose and clay-containing LbL coatings on foams [42,43]. Comparing the 3BL stress–strain curves, the assemblies produced with the three different GO grades appear almost overlapped, with the CHIT/GO_A_ foam being only slightly more rigid than the/GO_B_ and CHIT/GO_C_. The observed behavior at 3BL confirms the formation of assemblies characterized by ionic interaction densities that increase while moving from GO_A_ to GO_C_. This produces assemblies that are progressively stronger (from GO_A_ to GO_C_) and result in similar foam stiffness, even if the weight gain is considerably different (compare weight gain values in Table 2). The deposition of 6BL further increases stiffness, highlighting differences between the coatings embedding different GO grades, suggesting the coating thickness/weight gain to play a discriminating role only at 6BL. This trend is also reflected by the firmness values (Figure 7c), which represent the stress required to achieve a 40% compressive deformation and are associated with the comfort of the foam. The higher the value the more firmly the foam would behave. Commercially available open cell flexible PU foams normally show firmness values in the range of 2–6 kPa as a function of composition and density. The values obtained for LbL-modified foams in this work, ranging between 5 and 6.5 kPa, fall in the top range of commercial products and may therefore find application replacing high density (40–50 kg/m^3^) PU foams. This fact is of practical importance in transport applications. Indeed, the use of the foams reported in this paper with densities ranging from 22 to 24 kg/m^3^ would allow for an overall reduction in the weight ascribed to flexible foams.

## 4. Conclusions

This work evaluated the effects of different GO dimensions on the layer-by-layer assembly and properties of CHIT/GO coatings deposited on PU foams. The coating growth monitored by FTIR spectroscopy evidenced a linear assembly regime for all GO grades under study, highlighting that the thinnest nanoplates achieved the highest density of CHIT/GO interactions. Cross-sectional morphological observations confirm the dependence of the coating thickness from the GO thickness and the concentration of functional groups involved in the LbL assembly, highlighting a thickness coating trend as GO_A_ > GO_B_ > GO_C_, pointing out a direct relationship between the assembly thickness and GO dimensions. Either 3 or 6BL of each CHIT/GO assembly were successfully deposited on PU foams, yielding homogenous coatings capable of completely wrapping the complex PU 3D structure with weight gains proportional to the GO dimensions. The achieved flame-retardant properties were evaluated by means of horizontal flammability and forced combustion tests. All the LbL-treated foams were capable of completely suppressing the melt-dripping phenomenon. During flammability tests, the presence of the GO-based coatings extinguished the flame before the complete decomposition of the foam. This was demonstrated by the high residue values (70–80%) thanks to the high capability of GO nanoplates to act as a barrier to heat and mass exchange between the gas and the condensed phase. Forced combustion tests pointed out the ability of the CHIT/GO assembly to reduce, regardless of the GO dimensions, the heat release rates (pkHRR reduced by ~50%) and the total smoke release (TSR reduced by ~70%). However, a correlation with the different coatings weight gain allowed us to discriminate the assembly comprising the thinnest GO as the most efficient one. This was explained by the formation of a brick and mortar structure, capable of controlling the mass and heat exchange between the flame and the PU, thanks to the maximized nanoparticle overlapping. Compressive stress/strain mechanical tests demonstrated that the presence of the LbL coating increases the foam stiffness while maintaining the foam ability of recovering deformation after being cyclically compressed. The results further indicate that the through-thickness density of ionic interactions contributes the most to the increase in foam stiffness while the coating weight gain only plays a minor role at high BL numbers. The foams presented in this manuscript could find application in the transportation sector. In order to achieve this goal, future studies should focus on the evaluation of the coating durability to cleaning, abrasion and mechanical cycling.

In conclusion, the present study clearly points out the greater importance of achieving an optimal nanostructuration over the total coating weight gain. These results could potentially be extended to other nanoplatelets containing LbL coatings in order to develop advanced materials where the flame-retardant performances and the mechanical properties are optimized.

## Figures and Tables

**Table 1:** Viscosity and laser diffraction (LD) measurements conducted on GO_A_, GO_B_ and GO_C_ suspensions.

Sample	pH	Viscosity (mPa∙s)	D50 * (µm)
**GO_A_**	1.58 ± 0.08	10.8 ± 0.4	61 ± 2
**GO_B_**	1.51 ± 0.03	30.5 ± 0.7	39 ± 2
**GO_C_**	1.39 ± 0.06	77.5 ± 3.5	34 ± 3

* D50 is defined as the value of the particle diameter at 50% in the cumulative distribution, where half of the sizes are smaller than this value and half are larger.

**Table 2:** Weight gain, flammability and cone calorimetry results for neat and LbL-treated PU foams.

Sample		Flammability			Cone Calorimetry				
	Weight Gain (%)	Melt Dripping	Flame Spread Rate (mm/s)	Residue (%)	TTI ± σ (s)	pkHRR (kW/m^2^)	THR (MJ/m^2^)	TSR (m^2^/m^2^)	Residue (%)
**PU**		Yes	4.9 ± 0.4	-	3 ± 1	308 ± 25	10 ± 1	170 ± 11	7 ± 1
**3BL CHIT/GO_A_**	11 ± 2	No	7.3 ± 0.3	67 ± 1	2 ± 1	149 ± 3	9 ± 0.1	54 ± 1	11 ± 1
**3BL CHIT/GO_B_**	8 ± 1	No	6.5 ± 0.4	67 ± 1	2 ± 1	163 ± 19	10 ± 2	56 ± 12	10 ± 1
**3BL CHIT/GO_C_**	6 ± 1	No	5.9 ± 0.7	63 ± 1	2 ± 1	163 ± 11	9 ± 0.3	81 ± 10	8 ± 1
**6BL CHIT/GO_A_**	27 ± 2	No	4.2 ± 0.1	80 ± 1	2 ± 1	143 ± 4	9 ± 0.6	40 ± 3	12 ± 1
**6BL CHIT/GO_B_**	24 ± 2	No	4.3 ± 0.1	80 ± 1	2 ± 1	154 ± 9	11 ± 1.5	74 ± 27	12 ± 1
**6BL CHIT/GO_C_**	12 ± 1	No	5.5 ± 0.6	70 ± 1	2 ± 1	143 ± 7	10 ± 1	64 ± 8	11 ± 1

## Data Availability

The data presented in this study are available on request from the corresponding author.

## Supplementary Materials

The following are available online at https://www.mdpi.com/2079-4991/11/2/266/s1, Graphite oxide preparation and characterization; Figure S1 FTIR spectra of Chitosan and GO_A_ signal attribution; Table S1 Attribution of chitosan and graphite oxide FT-IR signal; Figure S2 FTIR LbL growth of 10BL CHIT/GO_A_ (a), 10BL CHIT/GO_B_ (b) and 10 BL CHIT/GO_C_ (c); Figure S3 Pictures of flammability test in horizontal configuration of 6BL CHIT/GO_A_ treated PU foam, 6BL CHIT/GO_B_ PU foam and 6BL CHIT/GO_C_ PU foam. First column: right after ignition, second column: 15 s after ignition and third column: end of the test.
