# Supplementary material for: Effects of Graphite Oxide Nanoparticle Size on the Functional Properties of Layer-by-Layer Coated Flexible Foams
Source: Nanomaterials (Basel). 2021 Jan 20;11(2):266. doi: 10.3390/nano11020266 (PMC7909570; doi:10.3390/nano11020266)
Supplement: Supplementary file 1 [file nanomaterials-11-00266-s001.pdf]

nanomaterials

Supplementary Materials

Effects of Graphite Oxide Nanoparticle Size on the Functional Properties of Layer-by-Layer Coated Flexible Foams

Lorenza Maddalena [1], Julio Gomez [2], Alberto Fina [1] and Federico Carosio [1,*]

[1] Dipartimento di Scienza Applicata e Tecnologia, Politecnico di Torino, Alessandria Campus, Viale Teresa Michel 5, 15121 Alessandria, Italy; lorenza.maddalena@polito.it (L.M.); alberto.fina@polito.it (A.F.)
[2] Avanzare Innovacion Tecnologica S.L, Avda. Lentiscares 4-6, Poligono Industrial Lentiscares, 26370 Navarrete, Spain; julio@avanzare.es
* Correspondence: federico.carosio@polito.it

Graphite oxide preparation and characterization

Water dispersion of graphite oxide was prepared using a modified Hummers' method in H_2SO_4. Starting from large flakes of natural graphite (NGS-Naturgraphit) and using a proportion of graphite/$KMnO_4$/$NaNO_3$ 1:3.75:0.25. The reaction temperature inside the reactor was kept between 0 and 4 °C during the oxidants addition (72 h). After that time, resulting solution was slowly warmed up to 20°C and maintained for 72 hours of reaction. To remove the excess of MnO_4^-, H_2O_2 solution was added to the reaction mixture and stirred overnight. After sedimentation, the solution was washed with a mechanical stirred HCl 4 %wt solution by 2 h. The solid was filtered off obtaining wet graphite oxide. Wet graphite oxide was dispersed in deionized water (1 %wt based on dry graphene oxide) and stirred in a in a Dispermat LC75 using a cowless helix at 1000 rpms for 10 minutes and them at 20.000 rpms for 60 seconds. Then, this dispersion was ultrasonicated with a UP400S HIELCHER using a H22 sonotrode with 90% of amplitude and full cycle condition to exfoliate the graphite oxide for different times in order to obtain GO with different average lateral size: 30 min for GO_A, 60 min for GO_B and 120 min for GO_C. The pH of the suspensions was measured in the first 24h after its preparation with a calibrated pH meter.

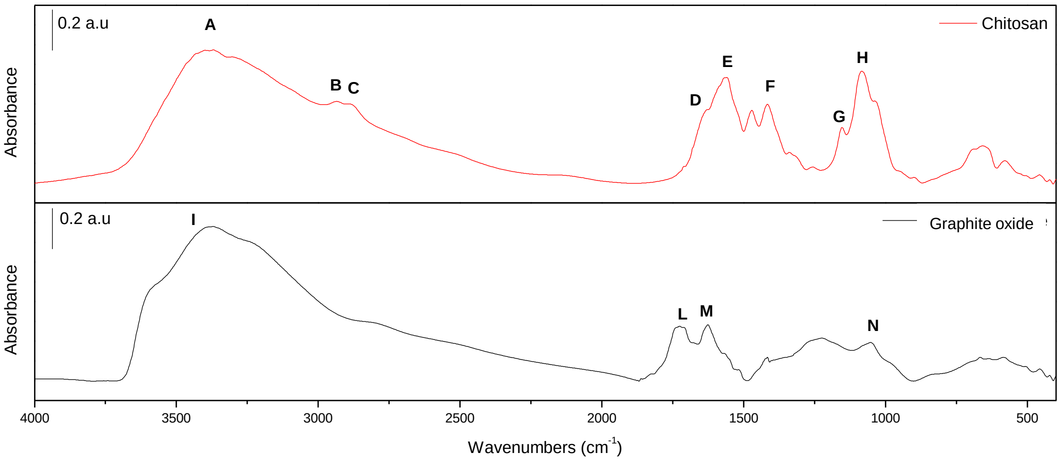

Figure S1. FTIR spectra of chitosan and GO_A signal attribution.

Table S1. Attribution of chitosan and graphite oxide FTIR signal.

	Signal	Wavenumbers (cm^{-1})	Attribution
Chitosan	A	3800–3000	ν(O-H) and ν(N-H)
	B	2900	ν_{as}(C-H) of CH_2
	C	2880	ν_s(C-H) of CH_2
	D	1640	δ_{as} (NH_3^+)
	E	1556	δ_s (NH_3^+)
	F	1156	NH_3^+ rocking
	G	1411	δ(C-H) of CH_2
	H	1080	ν(C-O-C) of glycosidic units
Graphite Oxide	I	3800–3000	ν(O-H)
	L	1725	ν(C=O)
	M	1627	ν($^-$O-C=O)
	N	1054	ν(C-O)

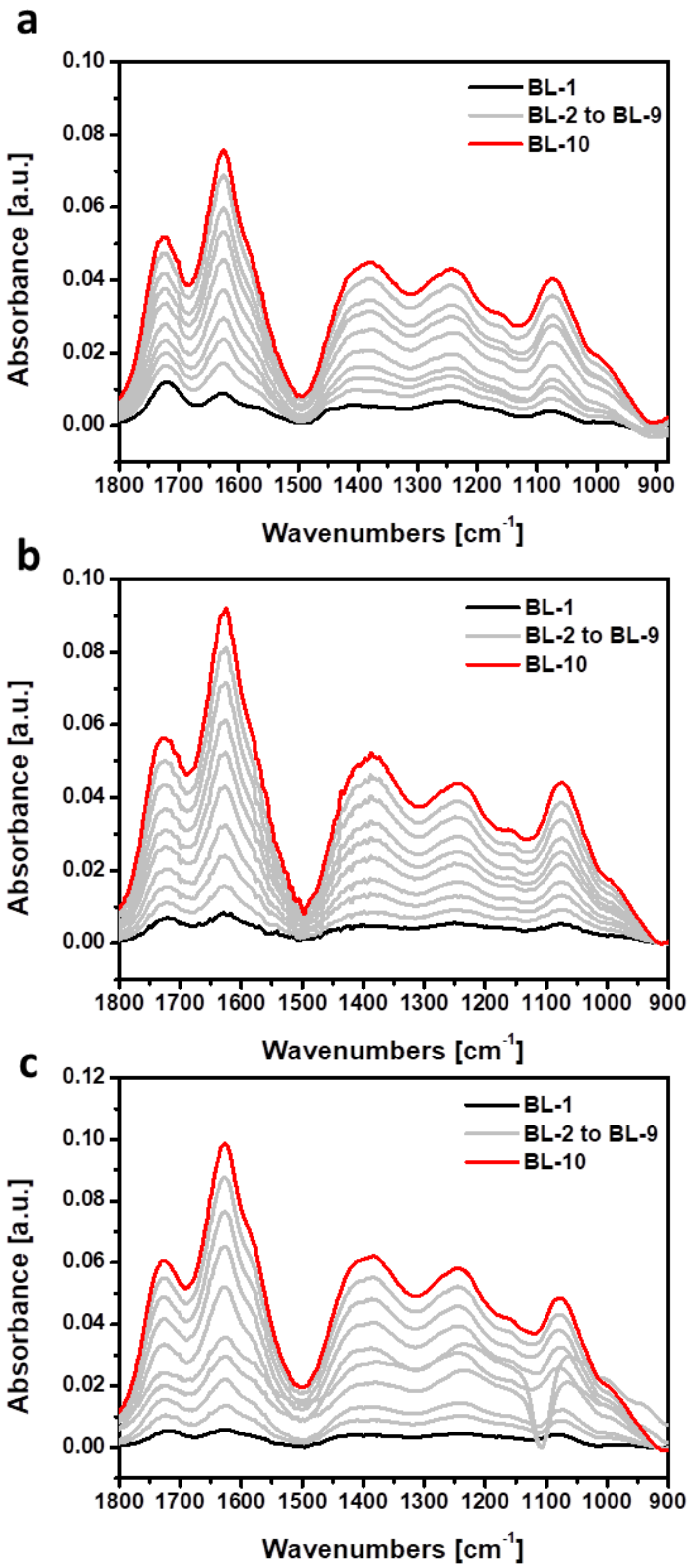

Figure S2. FTIR LbL growth of 10 BL CHIT/GO$_A$ (**a**), 10 BL CHIT/GO$_B$ (**b**) and 10 BL CHIT/GO$_C$ (**c**).

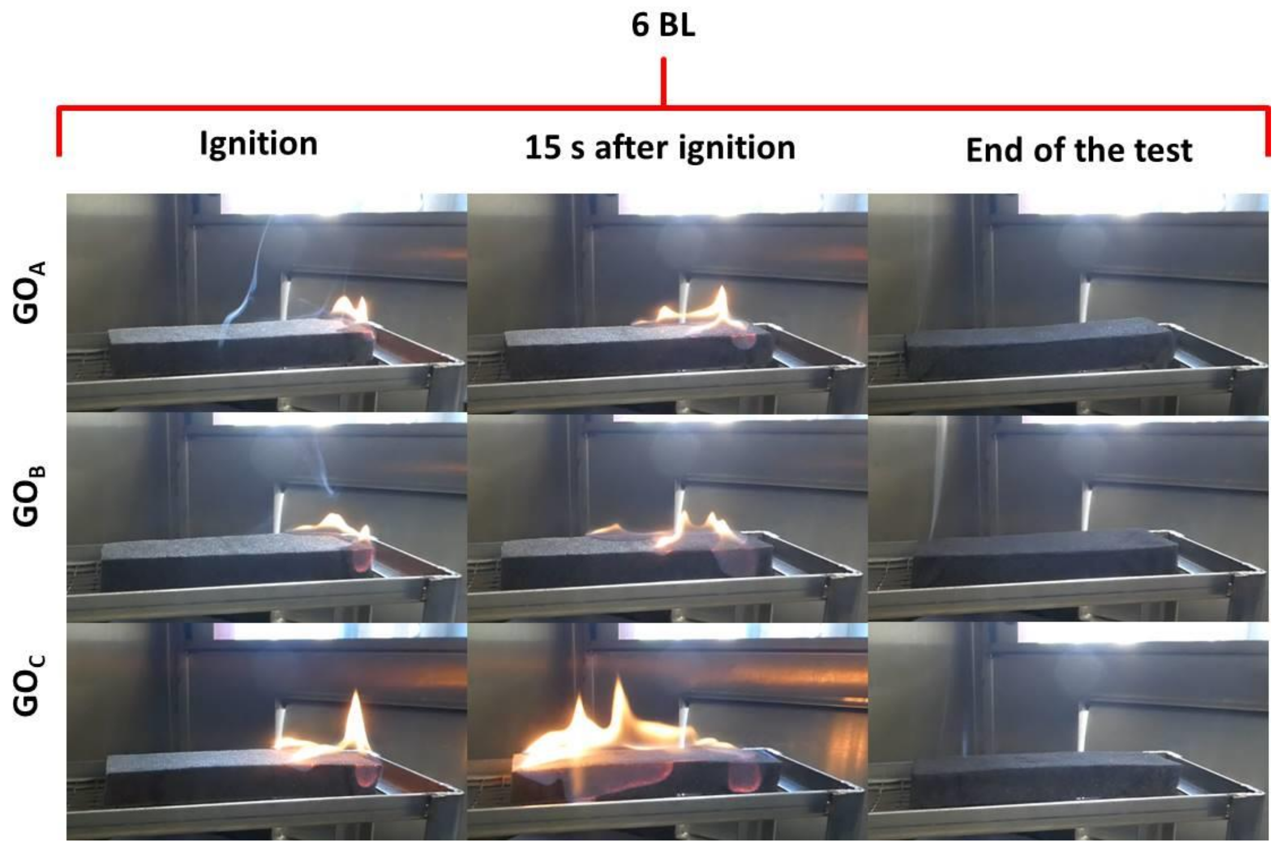

Figure S3. Pictures of flammability test in horizontal configuration of 6 BL CHIT/GO_A treated PU foam, 6 BL CHIT/GO_B PU foam and 6 BL CHIT/GO_C PU foam . First column: right after ignition, second column: 15 seconds after ignition and third column: end of the test.
